# Safety and preliminary efficacy of sequential multiple ascending doses of solnatide to treat pulmonary permeability edema in patients with moderate-to-severe ARDS—a randomized, placebo-controlled, double-blind trial

**DOI:** 10.1186/s13063-021-05588-9

**Published:** 2021-09-20

**Authors:** Benedikt Schmid, Markus Kredel, Roman Ullrich, Katharina Krenn, Rudolf Lucas, Klaus Markstaller, Bernhard Fischer, Peter Kranke, Patrick Meybohm, Bernhard Zwißler, Sandra Frank, and the Solnatide Collaborators Group

**Affiliations:** 1 Department of Anaesthesiology, Intensive Care, Emergency and Pain Medicine, University Hospital Wuerzburg, Wuerzburg, Germany; 2 Department of Anaesthesia, General Intensive Care and Pain Medicine, Medical University of Vienna, Vienna, Austria; 3 Vascular Biology Center, Division of Pulmonary Medicine, Medical College of Georgia, Augusta University, Augusta, USA; 4 Apeptico Forschung und Entwicklung GmbH, Vienna, Austria; 5 Department of Anesthesiology, University Hospital of Ludwig-Maximilians-University (LMU), Munich, Germany; 6 Comprehensive Pulmonary Center Munich (CPC-M), Member of the German Center for Lung Research (DZL), Munich, Germany

**Keywords:** Acute respiratory distress syndrome, Solnatide, Extravascular lung water, Pulmonary edema, Critical care

## Abstract

**Background:**

Acute respiratory distress syndrome (ARDS) is a complex clinical diagnosis with various possible etiologies. One common feature, however, is pulmonary permeability edema, which leads to an increased alveolar diffusion pathway and, subsequently, impaired oxygenation and decarboxylation. A novel inhaled peptide agent (AP301, solnatide) was shown to markedly reduce pulmonary edema in animal models of ARDS and to be safe to administer to healthy humans in a Phase I clinical trial. Here, we present the protocol for a Phase IIB clinical trial investigating the safety and possible future efficacy endpoints in ARDS patients.

**Methods:**

This is a randomized, placebo-controlled, double-blind intervention study. Patients with moderate to severe ARDS in need of mechanical ventilation will be randomized to parallel groups receiving escalating doses of solnatide or placebo, respectively. Before advancing to a higher dose, a data safety monitoring board will investigate the data from previous patients for any indication of patient safety violations. The intervention (application of the investigational drug) takes places twice daily over the course of 7 days, ensued by a follow-up period of another 21 days.

**Discussion:**

The patients to be included in this trial will be severely sick and in need of mechanical ventilation. The amount of data to be collected upon screening and during the course of the intervention phase is substantial and the potential timeframe for inclusion of any given patient is short. However, when prepared properly, adherence to this protocol will make for the acquisition of reliable data. Particular diligence needs to be exercised with respect to informed consent, because eligible patients will most likely be comatose and/or deeply sedated at the time of inclusion.

**Trial registration:**

This trial was prospectively registered with the EU Clinical trials register (clinicaltrialsregister.eu). EudraCT Number: 2017-003855-47.

## Administrative information

The order of the items has been modified to group similar items (see http://www.equator-network.org/reporting-guidelines/spirit-2013-statement-defining-standard-protocol-items-for-clinical-trials/).

**Table:** 

Title {1}	Safety and preliminary efficacy of sequential multiple ascending doses of solnatide to treat pulmonary permeability edema in patients with moderate-to-severe ARDS - a randomized, placebo-controlled, double-blind trial
Trial registration {2a and 2b}.	Primary Registry and trial identifying number: clinicaltrialsregister.eu ; EudraCT 2017-003855-47 Date of registration in primary registry: 2 February 2018 Secondary identifying numbers: not applicable Sources of monetary or material support: Apeptico Forschung und Entwicklung GmbH Primary sponsor: Apeptico Forschung und Entwicklung GmbH Secondary sponsor(s): not applicable Contact for public queries: Apeptico Forschung und Entwicklung Prof. Dr. Bernhard Fischer Mariahilfer Str. 136, Top 1.15 1150 Vienna, Austria 00436641432919 b.fischer@apeptico.com Contact for scientific queries: Apeptico Forschung und Entwicklung Prof. Dr. Bernhard Fischer Mariahilfer Str. 136, Top 1.15 1150 Vienna, Austria 00436641432919 b.fischer@apeptico.com Public title: Safety and preliminary efficacy of sequential multiple ascending doses of solnatide to treat pulmonary permeability edema in patients with moderate-to-severe ARDS - a randomized, placebo-controlled, double-blind trial Scientific title: Safety and preliminary efficacy of sequential multiple ascending doses of solnatide to treat pulmonary permeability edema in patients with moderate-to-severe ARDS - a randomized, placebo-controlled, double-blind trial Countries of recruitment: Austria, Germany Health conditions or problems studied: pulmonary permeability edema in patients with moderate-to-severe ARDS Interventions: Bronchial inhalation of ascending doses of solnatide vs. placebo for seven days Key inclusion and exclusion criteria: Ages eligible: ≥ 18 years Sexes eligible: both Accepts healthy volunteers: no Inclusion criteria: diagnosis of moderate to severe ARDS according to the Berlin definition, need for mechanical ventilation, pulmonary edema (EVLWI ≥10 ml/kg PBW), informed consent Exclusion criteria: allergy against solnatide, severe septic shock, extracorporeal membrane oxygenation at time of screening Study type: interventional allocation: randomized intervention model: parallel assignment masking: double blind (patient, caregiver, investigator, outcomes assessor) Date of first enrolment: 23.05.2018 Target sample size: 95 Recruitment status: ongoing Primary outcome: Safety (mortality, AEs, SAEs, laboratory data, ECG) Key secondary outcomes: extravascular lung water index (EVLWI), pulmonary vascular permeability index (PVPI), oxygenation ratio, ventilation parameters
Protocol version {3}	Version 8.0, 2 August 2021
Funding {4}	Apeptico Entwicklung und Forschung GmbH Commercial sponsor
Author details {5a}	BS, MK, PK, PM: Department of Anaesthesiology, Intensive Care, Emergency and Pain Medicine, University Hospital Wuerzburg, Wuerzburg, Germany RU, KK, KM: Department of Anaesthesia, General Intensive Care and Pain Medicine, Medical University of Vienna, Austria BF: Apeptico Forschung und Entwicklung GmbH, Vienna, Austria BZ, SF: Department of Anesthesiology, University Hospital of Ludwig-Maximilians-University (LMU), Munich, Germany RL: Vascular Biology Center, Division of Pulmonary Medicine, Medical College of Georgia, Augusta University, Augusta, USA
Name and contact information for the trial sponsor {5b}	Apeptico Forschung und Entwicklung GmbH Prof. Dr. Bernhard Fischer Mariahilfer Straße 136 1150 Vienna, Austria
Role of sponsor {5c}	The role of the sponsor was limited to the study design proposal, obtaining ethics and agencies approval, as well trial registration. The sponsor has no role in the management, analysis and interpretation of data, writing of the report, and the decision to submit the report for publication.

## Introduction

### Background and rationale {6a}

#### Acute respiratory distress syndrome (ARDS)

ARDS is a common pathology seen in intensive care units globally, with associated significant mortality and even long-term morbidity. ARDS is a clinical diagnosis accounting for up to 20% of unplanned ICU admissions. Mortality in ARDS patients is still high. The LUNG SAFE study [Belani2016] reports a hospital mortality of 40%, with a significant increase across the ARDS severity categories, in line with Berlin definition (34.9%, in mild ARDS; 40.3% in moderate ARDS; 46.1% in severe ARDS). The socioeconomic burden of the disease of critical illness is immense. A recent systematic review regarding the endpoint “return to work after critical illness” reported that approximately two-thirds, two-fifths, and one-third of previously employed intensive care unit survivors are jobless up to 3, 12, and 60 months following hospital discharge [1].

The most recent Berlin definition of ARDS [2] is shown in Table 1. The pathophysiology of ARDS is not completely understood. Initially, a direct pulmonary or indirect extrapulmonary insult is believed to cause a release of inflammatory mediators that promotes neutrophil accumulation in the microcirculation of the lung. Activated neutrophils migrate in large numbers across the vascular endothelial and alveolar epithelial surfaces and release proteases, cytokines, and reactive oxygen species (ROS). The migration and mediator release of neutrophils lead to pathologic vascular permeability, gaps in the alveolar epithelial barrier, and necrosis of type I and II alveolar cells, resulting in pulmonary edema, hyaline membrane formation, loss of surfactant and ultimately alveolar collapse, ventilation/perfusion mismatch, decrease of pulmonary compliance, and altered gas exchange. Subsequent infiltration of fibroblasts can lead to collagen deposition, fibrosis, and worsening disease [3].

**Table 1:** The Berlin definition of acute respiratory distress syndrome

Timing	Within 1 week of a known clinical insult or new or worsening respiratory symptoms
Chest imaging	Bilateral opacities—not fully explained by effusions, lobar/lung collapse or nodules ^a^
Origin of edema	Respiratory failure not fully explained by cardiac failure or fluid overload Need objective assessment (e.g., echocardiography) to exclude hydrostatic edema if no risk factor present
Oxygenation	
Mild	200 mmHg < P_aO2_/F_iO2_ ≤ 300 mmHg with PEEP or CPAP ≥ 5 cm H_2_O ^b, c^
Moderate	100 mmHg < P_aO2_/F_iO2_ ≤ 200 mmHg with PEEP ≥ 5 cm H_2_O
Severe	P_aO2_/F_iO2_ ≤ 100 mmHg with PEEP ≥ 5 cm H_2_O

Abbreviations: *CPAP*, continuous positive airway pressure; *F*_*iO2*_, fraction of inspired oxygen; *P*_*aO2*_, partial pressure of arterial oxygen; *PEEP*, positive end-expiratory pressure

^a^ Chest radiograph or computed tomography scan

^b^ If altitude is higher than 1000 m, the correction factor should be calculated as follows: [P_aO2_/F_iO2_ × (barometric pressure/760)]

^c^ This may be delivered noninvasively in the mild acute respiratory distress syndrome group

Most cases of ARDS in adults are associated with pulmonary sepsis (46%) or non-pulmonary sepsis (33%). Risk factors include those causing direct lung injury (e.g., pneumonia, inhalation injury, pulmonary contusion) and those causing indirect lung injury (e.g., non-pulmonary sepsis, burns, polytrauma, transfusion-related acute lung injury) [4].

ARDS is an orphan condition. The in-hospital mortality rate for ARDS is estimated to be between 34 and 55% [5]. Risk factors for mortality include older age, multiorgan dysfunction and presence of pulmonary and non-pulmonary comorbidities. Most ARDS-related deaths are due to multiorgan failure. Refractory hypoxemia accounts for only 16 percent of ARDS-related deaths [6].

Treatment of ARDS is supportive, including mechanical ventilation, prevention of stress ulcers and venous thromboembolism, nutritional support and treatment of the underlying disease. Pharmacologic options for the treatment of ARDS are limited. The use of corticosteroids is controversial; randomized controlled trials and cohort studies tend to support the early use of corticosteroids for decreasing the number of days on a ventilator; however, no consistent mortality benefit has been shown with this therapy [7, 8]. No targeted pharmacological treatment of patients with pulmonary permeability edema in ARDS is currently available, and therefore, new therapeutic approaches are warranted.

### Solnatide (INN, laboratory code AP301)—background and pre-clinical studies

Solnatide (AP301) is a synthetic peptide composed of 17 natural amino acids (Cys–Gly–Gln–Arg–Glu–Thr–Pro–Glu–Gly–Ala–Glu–Ala–LysPro–Trp–Tyr–Cys) with a molecular mass of about 2000 Da. Basically, solnatide is a circularized presentation of the lectin-like domain (so called TIP domain) of human TNF-α. Solnatide lacks any known pro-inflammatory activity to TNF-α. The Investigational Medicinal Product (IMP) “Solnatide 25 mg powder for reconstitution for solution for inhalation” is a sterile, lyophilized preparation of solnatide and contains no additional ingredients. The clinical route of administration is pulmonary delivery by inhalation of a liquid aerosol.

Pharmacological studies using rodent models have indicated that solnatide is an important regulator of alveolar fluid balance in healthy and injured lungs. Intratracheally and pulmonary-administered solnatide improved alveolar liquid clearance (ALC) in in situ flooded mouse lungs, in ex vivo models of flooded rat lungs, and in rat lungs prior to transplantation [9–11]. In a porcine bronchoalveolar lavage model of ARDS, inhalation of nebulized solnatide resulted in an increased P_a_O_2_ /F_i_O_2_ ratio and reduced extravascular lung water (EVLW) [12].

The alveolar fluid clearing capacity of solnatide is related to activation of the amiloride-sensitive sodium channel (ENaC), the major driving force for reabsorption of water through the alveolar epithelium [13–17]. ENaC is responsible for the maintenance of Na^+^ balance, extracellular fluid volume, and blood pressure and is located at the apical membrane of salt-reabsorbing tight epithelia of the distal nephron, the distal colon, salivary and sweat glands, and the lung, where it constitutes the rate-limiting step for vectorial movement of Na^+^ ions from the luminal side into the cell interior. The basolaterally located Na-K-ATPase actively transports Na^+^ out of the cell, providing the driving force for Na^+^ reabsorption. In the lung, Na^+^ transport through apically located ENaC in the alveolar epithelium is crucial for maintaining the correct composition and volume of alveolar lining fluid, enabling optimal gas exchange [18, 19]. Disruption of these processes occurs in pathologies in which permeability of the alveolar epithelium and pulmonary capillary endothelium is increased, leading to excessive accumulation of alveolar fluid and edema [20].

In addition to its ALC activity, solnatide inhibits hypoxia-induced reactive oxygen species (ROS) production and counteracts various ROS and toxin-mediated effects: solnatide inhibits protein kinase C (PKC) alpha activation thereby restoring ENaC activity. Solnatide reduces the degree of myosin light chain (MLC) phosphorylation and thus protects and restores the barrier integrity of endothelial and epithelial cells [21]. Solnatide lacks pro-inflammatory activity and does not lead to an increased production of chemokines or an increased infiltration of neutrophils in rat lungs upon intratracheal instillation.

The standard battery of safety pharmacology studies did not reveal drug-related adverse effects in any of the animal models. From animal studies in various species (rodent and non-rodent), it is evident that peptide clearance from blood is quite fast (decrease by a magnitude of 3 orders within 2 h) after bolus intravenous injection of 25 mg solnatide/kg BW. In the 14-day toxicity study in the rat, lung tissue samples were taken after the last treatment on day 14 and analyzed for the presence of solnatide; no test item was detected in lung tissue approximately 30, 60, and 90 min after the end of treatment in the low-, mid-, and high-dose group respectively.

### Solnatide (AP301)—clinical studies

The safety, tolerability and preliminary efficacy of solnatide have been assessed in three placebo-controlled, randomized, double-blinded clinical studies.

Study 2011-000223-33 was a “first in man” dose escalation study in healthy male subjects (*N* = 48; 36 vs 12) to investigate the safety, tolerability, and systemic exposure of orally inhaled single doses of solnatide. The highest tested dose was 171.4 mg per subject (nebulizer filling dose), which corresponds approximately to the no observed adverse effect level (NOAEL) dose of the sub-chronic toxicity (14 days) study in the beagle dog. The study concluded that doses of up to 171.4 mg were safe and well tolerated. Consistent with pre-clinical data in various species, the distribution of inhaled solnatide was largely confined to the lung, as indicated by very low maximum (C_max_) and total (AUC_0_t_) solnatide systemic exposure levels. None of the comprehensive safety assessments employed (spirometry, quantification of exhaled NO, blood pressure, heart rate, ECG, safety laboratory variables, adverse event reports) indicated any clinically meaningful or remarkable dose- or time-related alterations of safety outcomes [22].

Study 2012-001863-64 was a proof of concept study in male and female intensive care patients (*N* = 40; 1:1) that investigated the clinical effect of repeated orally inhaled doses of solnatide on alveolar liquid clearance in acute lung injury. For safety reasons and according to effective pharmacological doses observed in various animal models, the second highest dose of the Phase I clinical study was chosen: 125 mg solnatide (nebulizer filling dose) twice daily (i.e., every 12 h) delivered per endotracheal inhalation to each subject. The main efficacy outcome was a positive trend in pulmonary edema (EVLWI) reduction in the whole study population, which is significant and more pronounced in patients with an initial Sequential Organ Failure Assessment (SOFA) score above 10 inhaling solnatide versus placebo (*P* = 0.04; exploratory subgroup analysis). The secondary endpoint analysis revealed a trend towards an earlier improvement of peak, plateau and mean airway pressure, PEEP, and Murray lung injury score in patients treated with solnatide. This effect was again most prominent in patients with an initial SOFA score > 10 (exploratory subgroup analysis). In addition, a shorter duration of mechanical ventilation and increased number of ventilator-free days in patients receiving solnatide inhalations versus placebo was observed, which again was more pronounced in patients with initial SOFA score above 10 (*P* = 0.06; exploratory subgroup analysis). No significant differences in the occurrence of adverse events and severe adverse events were found between the study groups that could not be explained by the underlying diseases [23].

Study 2013-000716-21 was a pilot study that investigated the clinical effect of orally inhaled solnatide on treatment of primary graft dysfunction (PGD) in mechanically ventilated patients (*N* = 20; 1:1) after primary lung transplantation. When compared to placebo, oral inhalation of solnatide in patients with PGD following lung transplantation led to earlier improvement of gas exchange, reduction of EVLWI, earlier extubation / shorter mechanical ventilation, a shorter stay at the ICU, and earlier discharge from the hospital. No drug-related adverse events, drug-related serious adverse events, or deaths were reported.

Inhalation of solnatide provides a novel therapy to increase alveolar fluid clearance directly, with the potential to decrease ventilatory pressures earlier and to improve weaning from mechanical ventilation. The main cause of death in critically ill patients is underlying disease and organ failure. Nevertheless, an improvement in pulmonary function may lead to improvement in important outcome parameters including length of mechanical ventilation and ICU stay, hospital stay, morbidity, and mortality.

### Known potential risks

Solnatide is generally well tolerated. No deaths or serious adverse events occurred during the Phase I study [22]. Only five possibly solnatide-related adverse events (AEs) were found in four of the 48 subjects, but all five were deemed mild and transient by the investigator, did not require any medical intervention, and were resolved spontaneously. The cause of the AEs (flatulence, abdominal pain, hiccups, and headache) was likely related to procedural aspects, as the constant presence of the investigator, the requirement for continuous uninterrupted oral inhalation using nose-clips for several minutes and the large number of examinations during the period of observation (e.g., frequent drawing of blood samples or measurement of lung function) could have easily led to stress and stress-related symptoms in some subjects. “Headache” was generally the most common adverse event in Phase I trials in healthy subjects and is generally accepted to be due to the dietary restrictions such as caffeine withdrawal [24]. A mild decrease of leucocytes in one subject could have been due to a congenital disorder of blood cells and leucocytes, which spontaneously returned to normal range after 20 days without treatment. No AE led to any subject being withdrawn from the study. No significant effect of solnatide on vital signs was detected during the study. Given that patients with ARDS are sedated and under mechanical ventilation, the stress-related AEs mentioned above are unlikely to occur and would in any case be insignificant and pose little risk considering the clinical condition of the patients.

Solnatide was quantifiable in plasma only at very low concentrations (i.e., < 2.5 ng/ml) and for a brief period shortly after inhalation. Systemic bioavailability is extremely low, so no significant systemic effects are expected.

During the Phase II proof of concept study, 96 AEs occurred during the first week of therapy and 59 AEs occurred within the following 3 weeks of observation. The most frequent AEs are common conditions in critically ill patients with acute lung injury and many AEs were related to the underlying diseases. Other AEs were related to comorbidities. In the opinion of the investigators, all AEs were unrelated to study therapy with one exception: A decreased tidal volume on pressure-controlled ventilation occurring immediately after inhalation of solnatide in a 67-year-old male patient with 60% burn injuries was the only possible treatment-associated AE that could be identified. After bronchoscopy and treatment with inhaled bronchodilators and steroids, the patient was weaned to assisted ventilation on the same day and did not exhibit any adverse reactions to subsequent solnatide treatment.

Currently, there is not enough data available to consider any event to be expected for the purpose of regulatory reporting.

Hemodynamic monitoring with systems such as PiCCO® from Pulsion or VolumeView from Edwards used in this study requires the application of a central venous catheter and an arterial catheter via the femoral artery. Femoral cannulation replaces the routine radial cannulation used for hemodynamic monitoring. The procedure is therefore invasive and complications may occur, although infrequently. A study by Scheer et al. identified 11 studies that used the femoral artery for hemodynamic monitoring. Temporary occlusion was reported in 10 patients (mean incidence 1.45%), and serious ischemic complications requiring extremity amputation was reported in three patients (0.18%). Pseudoaneurysm formation occurred in 6 patients (0.3%), sepsis in 13 patients (0.44%), and local infection in 5 patients (0.78%). Bleeding (generally minor) was observed in 5 patients (1.58%), and hematoma formation in 28 (6.1%). One patient developed an infected hematoma and needed blood transfusion and another patient eventually died from massive retroperitoneal bleeding. On the basis of this systematic review, the authors concluded that serious complications of the radial, femoral, and axillary artery are rare and that arterial cannulation is a relatively safe procedure [25].

### Known potential benefits

Application of solnatide directly into the lower respiratory tract, in the form of a liquid aerosol, is expected to activate the pulmonary sodium ion channel (ENaC) to directly activate alveolar liquid clearance and to reduce the leakage of blood and fluids from the capillaries in the airspace, i.e., accelerate the resolution of alveolar edema and reduce barrier injury in the lung.

Inhalation of nebulized solnatide by patients with pulmonary edema and ARDS resulted in a reduction of EVLWI as well as a trend towards a higher number of ventilator-free days in a subgroup of patients with an initial SOFA score above 10 [23].

In numerous clinical studies, the correlation between the amount of pulmonary edema (determined as EVLWI) and outcome parameters in patients with ARDS has been examined. In these studies, a strong correlation between elevated pulmonary edema and a worse outcome has been demonstrated.

Taken together, the alveolar liquid clearance activity of solnatide as well as its counteracting activity against various ROS and toxin-mediated effects is expected to support the alveolar repair process and accelerate restoration of function. In consequence, this should lead to reduced airway pressures, increased oxygenation, and eventually shortened duration of assisted ventilation. This will limit further lung injury and prevent ventilator-associated lung injury as well as improve oxygenation of vital organs. Both aspects are of importance as the volume of aerated lung is reduced in patients with ARDS and normal tidal volumes delivered with airway pressures that are considered safe for the uninjured lung may cause regional overdistention. Shortening the duration of assisted ventilation also limits atelectrauma and biotrauma.

A rapid improvement of lung function may also lead to decreased treatment days at ICU and positively influence survival. However, due to the multifactorial nature of ARDS, a reduction of mortality can only be established by a balanced regime of identification and successful treatment of the underlying cause (or causes), supportive therapy limiting further lung injury, and specific therapy reducing lung injury. Nevertheless, a rapid improvement of pulmonary function is an important pre-requisite for improved clinical outcomes in patients with pulmonary permeability edema / ARDS who have a clinically “manageable / curable” primary disease / condition.

### Assessment of potential risks and benefits

Given the good safety and tolerability profile so far demonstrated in the clinical development program of solnatide and, given its potential therapeutic effect in life-threatening pulmonary edema, the benefits of this trial are expected to outweigh its potential risks.

This Phase IIB trial follows a risk-adjusted procedure by utilizing a dose escalation scheme of 5 mg, 60 mg and 125 mg solnatide per single administration (nebulizer filling dose). Escalation from one dose to the next will occur only once a Data Safety Monitoring Board (DSMB) has carefully reviewed the safety data emerging from the last treatment group and has approved the administration of the next highest dosage. In contrast to the previous Phase I study in healthy male volunteers, this study will investigate safety and preliminary efficacy in patients with pulmonary permeability edema and ARDS.

In this Phase II study, seriously ill patients, already on ventilation, will receive the IMP on top of standard therapy directly over the ventilation system. These circumstances and the fact that examinations such as lung function measurements or blood samplings will be performed with the patient under sedation, mean that procedure-related AEs are unlikely to occur. In addition, assessment of EVLWI is a common procedure in patients with ARDS and is usually well tolerated.

ARDS patients in this study are under mechanical ventilation and most likely not able to give their informed consent prior to enrolment. However, given the noteworthy potential benefits and limited risks of the study, participation should not be precluded because of the sedated/unconscious state of the patient if the investigator and IRB/IEC believe that it is of potential benefit to and in the interest of the individual subject. Patients will be asked to provide consent once they are fit to do so.

Given the patient’s conditions and the good safety profile of solnatide, study procedures will not place any additional burden on the patient, nor should they create any foreseeable risks. The patients will nevertheless be closely and constantly monitored to ensure their health, safety, and wellbeing.

### Aim of the study

The aim of this prospective, randomized, controlled trial is to identify safe doses of solnatide for inhalative administration in ARDS patients.

## Objectives {7}

The main objective is to assess the local and systemic safety of 7 days orally inhaled sequential multiple ascending doses of solnatide in patients with pulmonary permeability edema and moderate-to-severe ARDS.

Secondary objective is to review potential efficacy endpoints for a future Phase III pivotal trial.

## Trial design {8}

This is a Phase IIb, randomized, placebo-controlled, double-blind, dose escalation study. The study will be conducted in up to 10 centers located in Germany and Austria. Additional European countries may be involved.

Thirty eligible patients with pulmonary permeability edema (EVLWI ≥ 10 ml/kg PBW) and moderate to severe ARDS admitted to an intensive care unit (ICU) and under mechanical ventilation will be randomized in a 2:1 ratio to receive either a low dose of solnatide (5 mg) (20 patients) or placebo (10 patients). Patients will be treated for up to 7 days and the study duration for each patient is of up to 28 days.

Following the end of the study period for these patients and prior to escalating from the low dose to the middle dose (60 mg), safety data collected during the 28-day study period will be reviewed by an independent Data Safety Monitoring Board (DSMB), who will decide whether the safety profile is acceptable for dose escalation. In case of positive outcome, 25 new patients will be randomized to solnatide 60 mg or placebo in a 4:1 ratio in order to have 20 patients randomized to the middle-dose group and 5 to the placebo group.

After the end of the study period of these patients and prior to escalating from the middle dose to the high dose (125 mg), safety data will again be reviewed by the DSMB, who will decide whether the safety profile is acceptable for escalating to the high dose.

If so, 40 new patients will be randomized to solnatide 125 mg and placebo in a 1:1 ratio in order to have 20 patients randomized to the high-dose group and 20 patients to the placebo group.

The randomization ratios solnatide/placebo (i.e. 2:1; 4:1; 1:1) will allow to have a total of 35 patients assigned to placebo. In addition, the randomisation schedule will provide the DSMB with enough placebo patients to perform the safety analysis.

**Fig. 1:**
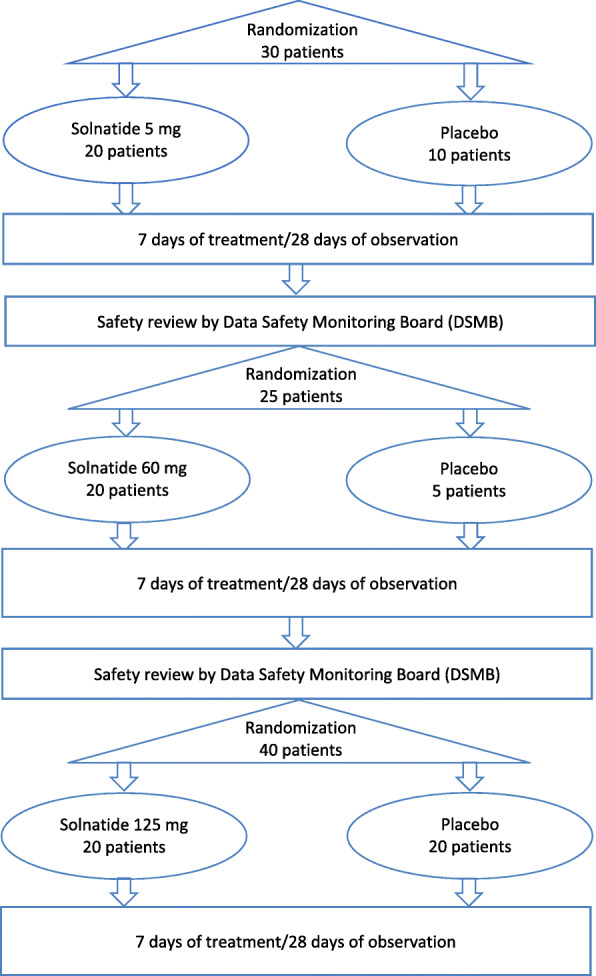
Trial design flowchart

To conduct the study, approximately 95 patients will therefore be randomized (see Fig. 1).

## Methods: participants, interventions and outcomes

### Study setting {9}

Patients from 9 academic hospitals in Austria and Germany will be eligible for enrolment. A list of study sites can be obtained on request from the Sponsor. Participating study centers will host intensive care units specialized in the treatment of moderate and severe forms of ARDS. Every ARDS patient will be evaluated for eligibility to participate in this study.

### Eligibility criteria {10}

#### Inclusion criteria

The study will enroll patients with pulmonary permeability edema (EVLWI ≥ 10 ml/kg PBW) and moderate to severe ARDS admitted to an intensive care unit and under mechanical ventilation. To be eligible to participate in this study, an individual must meet all the following criteria:

##### Informed consent

1. Male or female ≥ 18 years of age
2. Patient has been admitted to an ICU, is mechanically ventilated and stable in this condition for at least 8 h.
3. Moderate-to-severe ARDS diagnosis as defined by the Berlin definition:

- Onset of ARDS within 1 week of a known clinical insult or new or worsening respiratory symptoms.
- Bilateral opacities not fully explained by effusions, lobar/lung collapse, or nodules.
- Respiratory failure not fully explained by cardiac failure or fluid overload (origin of edema).
- PaO2/FiO2 ≤ 200 mm Hg with Positive End-Expiratory Pressure (PEEP) ≥ 5 cm H_2_O.

4. Verified ARDS diagnosis (moderate or severe according to Berlin definition) not older than 48 h.
5. Extravascular lung water index (EVLWI) ≥ 10 ml/kg PBW as assessed with a validated bedside measurement (single indicator transpulmonary thermodilution measurement, such as with PiCCO® from Pulsion or VolumeView from Edwards).
6. Patient who meets criteria for extensive hemodynamic monitoring as per international intensive care medicine standards.

#### Exclusion criteria

Patients must meet none of the exclusion criteria listed below.

1. History of clinically relevant allergies or idiosyncrasies to solnatide.
2. Known use of any other investigational or non-registered drug within 30 days or within 5 half-lives of these drugs prior to study enrolment, whichever is longer. No exceptions are allowed in this study.
3. Severe state of septic shock with a mean arterial pressure (MAP) ≤ 65 mmHg and a serum lactate level > 4 mmol/L (36 mg/dl) despite adequate volume resuscitation.
4. An underlying clinical condition that, in the opinion of the investigator, would make it very unlikely for the patient to be successfully weaned from ventilation due to severe underlying diseases (e.g., severe malnutrition, severe neurological diseases, pulmonary fibrosis, or COPD).
5. Extracorporeal membrane oxygenation, high-frequency oscillatory ventilation or any form of extracorporeal lung support. In no way are patients to be denied or delayed these procedures to avoid exclusion from the study.
6. Neutrophil count < 0.3 × 10^9^/L.
7. Cancer treatment (chemotherapy or biological) or therapy with other immunosuppressive agents for organ transplantation within 2 weeks.
8. Cachexia (BMI < 18.5 kg/m^2^).
9. Unequivocal cases of cardiogenic pulmonary edema (if differential diagnosis based on an ARDS triggering condition and clinical replicability is not possible, echocardiography may be indicated).
10. Severe skin burns involving more than 15% of body surface.
11. Subjects who are extremely unlikely to survive more than 48 h due to the acute conditions of the patient in the opinion of the investigator.
12. Subjects transferred from a hospital not participating in this study who are already planned to be re- transferred during the observation period.
13. Subjects who are not expected to survive the next month because of an underlying uncorrectable medical condition or a do not resuscitate order.
14. Women known to be pregnant, women who are lactating, women with a positive or indeterminate pregnancy test, on screening, and males of reproductive potential and women of childbearing potential who are not willing to use highly effective methods of birth control/contraception for a duration defined in the patient’s Informed Consent Form.
15. After randomization, drop-out of a participant is not envisaged by the protocol except for withdrawal of consent by the participant or his/her legal representative, discontinuation of the treatment (e.g., due to toxicity, or death of the patient during the treatment course).

### Who will take informed consent? {26a}

Informed consent will be taken by appointed investigators, who had been trained beforehand according to GCP standards. Consent forms describing in detail the study intervention, study procedures, and risks are given to the participant/legal representative, and written documentation of informed consent is required.

Since ARDS patients in this study are in the ICU under mechanical ventilation, it is very likely that most will not be able to give their written informed consent prior to enrolment in the study.

According to German Law persons unable to give consent are subject to specific legal regulations and therefore can be included into medical research projects only under strict requirements. To obtain consent the following procedures must be followed:

In case of an emergency, the investigator has to find out whether a patient’s representative has been appointed by power of attorney for personal care (“Vorsorgevollmacht”). Usually, this will be done by on-site interviewing of accompanying persons of the patient.

If a patient’s representative appointed by power of attorney for personal care (“Vorsorgevollmacht”) is available, he/she will check whether a patient’s decree (“Patientenverfügung”) exists and whether it is in accordance with the patient’s current living and treatment conditions. If a patient’s decree is available, the patient’s representative will make sure that the patient’s specifications will be implemented. If a patient’s decree is not available or is not in accordance with the specific situation, the patient’s representative is obliged to act according to the presumed patient’s will based upon oral or written statements, values, and ethical or religious beliefs of the patient concerned. To determine the presumed will of the patient whether to participate in a clinical study or not, close relatives, and other reliable persons might be involved provided that this process does not lead to any delays being in conflict with the urgency of the treatment.

The information and consent process will be carried out with the patient’s representative in the same way as with the patient. The patient’s representative will sign the consent form on behalf of the patient. If a patient is unable to give consent but still able to follow the information and consent process partially, the patient must also be informed. Even if expressed non-verbally, a patient’s wish either to participate or not to participate in the trial must be respected.

If a patient’s representative cannot be asked for consent to include the patient in the study, the appointment of a provisional patient’s representative must be made by the local district court (“Amtsgericht”). If, in cases of urgency, such an appointment is not possible, a judge may also take the decision for the benefit of the patient.

If neither the appointment of a patient’s representative nor a judicial urgent decision is possible in time, § 41 paragraph 1 clause 2 of the German Drug Law is applicable. According to this paragraph, “treatment which is necessary without delay to save the life of the person concerned, restore good health or alleviate suffering, can be dispensed immediately. Consent for continued participation must be obtained as soon as it is possible and reasonable”. In this case, the investigator must consider all known indications regarding the patient’s presumed will. It is the responsibility of the investigator to examine whether, in his view, the patient could be included in the clinical trial.

The process to determine the patient’s presumed will to participate in a clinical study will be documented in accordance with local procedures. Once the patient regains his/her ability to consent, he/she shall be asked to give his/her consent to the continuation of the clinical trial.

### Additional consent provisions for collection and use of participant data and biological specimens {26b}

Not applicable, biological samples will not be retained after the study is complete.

## Interventions

### Explanation for the choice of comparators {6b}

This ascending dose study consists in the sequential administration of 5 mg, 60 mg, and 125 mg of solnatide.

In cell-based non-clinical pharmacodynamic studies, the EC_100_ of solnatide-induced ENaC activation was found to be approximately 120 nM. Various models taking into account lung volume, lung weight, and lung surface area have indicated that a nebulizer filling dose of 5 mg solnatide corresponds most closely with the non-clinical cell-based experimental setup. A single application of 5 mg solnatide corresponds to the lowest dose of the previous Phase I clinical study (EudraCT: 2011-000223-33).

A bridging dose of 60 mg was chosen between the low and high dose. This dose was used in a pharmacological study in pigs and may be effective in a clinical setting also. In addition, this dose corresponds to the intermediate dose level of the previous Phase I clinical study (EudraCT: 2011-000223-33).

In previous clinical Phase II trials, patients received two daily doses each of 125 mg solnatide reconstituted in 5 ml water for injection delivered as aerosol via nebulization for up to 7 days. This dose level is below the highest dose level used in the previous Phase I study (EudraCT: 2011-000223-33). It represents a dose level corresponding to the most effective dose levels used in previous pharmacologic studies and showed promising outcomes in the two previous Phase IIa trials (EudraCT: 2012-001863-64; 2013- 000716-21).

### Intervention description {11a}

#### Active agent

The Investigational Medicinal Product (IMP) is “Solnatide 25 mg powder for reconstitution for solution for inhalation.” The IMP is a sterile lyophilized preparation of the drug substance solnatide, a synthetic peptide composed of 17 naturally occurring amino acids. One N-terminal cysteine and one C-terminal cysteine form an intra-molecular disulfide bridge. Solnatide contains no post-translational modifications.

Solnatide was manufactured, characterized, and released based on requirements of the European Pharmacopoeia, European and international quality and safety guidelines including ICH guidelines related to chemical compounds and / or synthetic peptides. Guidelines and regulations related to recombinant proteins do not apply to the development compound.

Prior to clinical use, each vial with the IMP is to be reconstituted with 1 ml of water for injection (commercial product) resulting in a solnatide solution with a concentration of 25 mg/ml. Reconstituted IMP in the required clinical-use concentration is provided as a clear solution in a closed single-use sterile plastic syringe for transportation purpose at 2–8 °C for immediate use with the nebulizer Aeroneb Solo.

However, in-use storage time of the reconstituted solution in the single-use plastic syringe may be extended up to 3 days at 2 to 8 °C, if required and a corresponding Manufacturer’s Authorization is in place.

The in-use stability of the reconstituted solution is described in the investigators’ brochure. The chemical and physical in-use stability of the reconstituted solution in the single-use plastic syringe has been demonstrated for 7 days at 2–8 °C. Sterility of the reconstituted solution in the single-use plastic syringe has been demonstrated after in-use storage times of 4 days at 2 to 8 °C provided reconstitution has taken place in controlled aseptic conditions.

#### Placebo

This is commercially available 0.9% saline solution in vials.

Patients will receive 5 ml of the study drug containing 5 mg, 60 mg, or 125 mg solnatide or 0.9% saline according to treatment allocation via endotracheal nebulization every 12 h for a maximum of 7 days using an Aeroneb Solo nebulizer.

Reconstituted solnatide as well as placebo solution (saline) will be supplied in a closed and labelled syringe to study personnel authorized for administration. Both solutions are clear and colorless, and thus indistinguishable in appearance.

#### Dosing and administration

In this dose ascending study, 3 groups of patients (one for each dosage) will be randomized to treatment with either solnatide or placebo. The three doses administered in this study are 5 mg, 60 mg, and 125 mg and escalation from one dose to the next will occur only once a DSMB has carefully reviewed the safety data emerging from the last treatment group and has approved the administration of the next highest dosage. The highest dosage used in this study has already been tested and has proved to be safe in a Phase I and two Phase II trials and no dose-limiting effects are expected.

Patients will be treated with solnatide or placebo twice a day for 7 days via endotracheal inhalation. Inhalations should be scheduled around 08:00 a.m. (± 30 min) and 08:00 p.m. (± 30 min). The 12-h sequence should be followed as closely as possible. If applicable to the routine of the ICU, inhalation can be shifted to a later time until 10:00 a.m. (± 30 min) and 10:00 p.m. (± 30 min), respectively, at the latest.

To enable endotracheal inhalation, reconstituted solnatide in water for injection is converted into an inhalable aerosol by the Aeroneb Solo medicinal device. This device is a product of Aerogen, Galway, Ireland, and is a commercially available liquid nebulizer. The Aerogen Solo nebulizer has been approved in the European Community by CE-marking based on requirements of Annex II, Section 3.2. of Directive 93/42/EEC.

The nebulizer unit holds up to 6 ml of liquid medication. The nebulizer unit is clear to allow visual monitoring of medication levels and aerosolization. When the nebulizer unit is connected to the ventilator circuit, the silicon plug can be opened and closed in between doses without causing loss of circuit pressure.

The T-piece of the nebulizer connects the unit into the breathing circuit via standard male and female 22-mm conical ports. The nebulizer unit together with the T-piece is placed into the inspiratory limb of the breathing circuit before the patient wye. Various in vitro studies and laboratory simulation have been conducted to characterize solnatide aerosol generation by the Aeroneb Solo nebulizer. Solnatide solution of 25 mg/ml showed effective nebulization and small condensation losses of solnatide aerosol in the connecting tube system. The nebulization time for 5 ml ranges between 10 and 20 min. Breath simulator studies showed that approx. 60 to 70% of nebulized solnatide was deposited on the inhalation filter (“lung”), indicating that the delivered dose of solnatide corresponds to about 60 to 70% of the nominal nebulizer filling dose.

### Criteria for discontinuing or modifying allocated interventions {11b}

Discontinuation from treatment does not mean discontinuation from the study, and remaining study procedures should be completed as indicated by the study protocol as described for any treatment day as well as follow-up period. If a clinically significant finding is identified (including, but not limited to changes from baseline) after enrolment, the investigator or qualified designee will determine if any change in participant management is needed. Any new clinically relevant finding will be reported as an adverse event (AE).

Every subject, the legal representative or authorized representative has the right to interrupt or to discontinue study participation at any time, for any reason, and every subject may be discontinued from the study for any reason beneficial to his/her wellbeing.

In addition, individual subjects must discontinue or withdraw from the study for any of the following reasons:

- Withdrawal of informed consent by the patient or the legal representative or authorized representative.
- Any AE, laboratory abnormality or intercurrent illness that, in the opinion of the investigator, indicates that continued participation in the study is not in the best interest of the subject.
- Suspected drug-related serious adverse event (SAE)
- Suspected unexpected serious adverse reaction (SUSAR)
- Allergies or idiosyncrasies to solnatide.

Patients withdrawn from the study will not be replaced with new participants.

If the cause of temporary withdrawal has been resolved, e.g., informed consent has been re-confirmed, a suspected drug-related SAE has not been confirmed by the investigator, a SUSAR has not been confirmed by the investigator, the patient may resume participation in the clinical study if the treatment schedule allows it.

Patients who are withdrawn from the study prematurely by the investigator or the sponsor will undergo all investigations as described for any treatment day for the end-of-study interview. Patients, the legal representative, or authorized representative who withdraw consent will be asked to accept further safety examinations in the interest of the patient.

### Strategies to improve adherence to interventions {11c}

The study medication will be administered only by authorized study personnel. Every effort will be made to ensure timely administration of the IMP as per protocol. If medication cannot be administered, the reason is to be documented in the eCRF and a protocol deviation recorded.

### Relevant concomitant care permitted or prohibited during the trial {11d}

There are no known interactions of solnatide with other substances. Accordingly, a general list of permitted and not-permitted concomitant medication is not available. In previous clinical studies, no drug- related SAEs and SUSARs have been observed.

As this is an add-on study in patients also receiving standard treatment, administration of concomitant medication is subject to the investigator’s decision and discretion and may vary for each individual patient due to the heterogeneity of ARDS and underlying etiologies.

### Provisions for post-trial care {30}

Specific post-trial care is not provided. Study participants are insured against any harm arising from the study interventions according to legal provisions.

### Outcomes {12}

The main objective of this study is to investigate the safety of solnatide administration. Therefore, no formal primary and secondary outcomes are defined. Safety will be assessed by a review of mortality, incidence of adverse events, and serious adverse events, as well as by analysis of relevant laboratory data and ECG. The following parameters will be taken into account when assessing the safety of solnatide:

- Vital signs (i.e., heart rate, systolic and diastolic blood pressure, and temperature)
- Clinical laboratory (i.e., white and red blood cell count, hematocrit, hemoglobin concentration, platelet count, creatinine, sodium, potassium, chloride, blood urea nitrogen, bilirubin, aspartate and alanine transaminases, lactate dehydrogenase, amylase, lipase, C-reactive protein, creatine kinase, arterial pH, PaO_2_, PaCO_2_, bicarbonate, SO_2_ as well as urine pH, specific weight, appearance, color, nitrites, proteins, glucose, urobilinogen, bilirubin, ketones, hematic pigments, and leukocytes)
- 12-lead electrocardiogram
- Hemodynamic parameters (i.e., mean arterial pressure, pulmonary blood volume, cardiac index, and cardiac output
- Need for vasoactive drugs
- 24-h fluid balance
- Concomitant medications
- All-cause deaths
- Utilization of extracorporeal treatments
- Adverse events and serious adverse events

The secondary objective of this study is to evaluate possible endpoints for a future Phase III pivotal trial. Such endpoints investigated in this study include:

- Extravascular lung water index
- Pulmonary vascular permeability index
- Murray lung injury score
- Oxygenation ratio
- Ventilation parameters (i.e., plateau pressure, tidal volume, positive end-expiratory pressure, peak inspiratory pressure, respiratory rate, F_i_O_2_, mean airway pressure, peak airway pressure, ventilation mode)
- Lung compliance
- Ventilation-free days
- Days of ICU
- Days of hospitalization
- Driving pressure
- Sequential organ failure assessment

### Participant timeline {13}

**Fig. 2:**
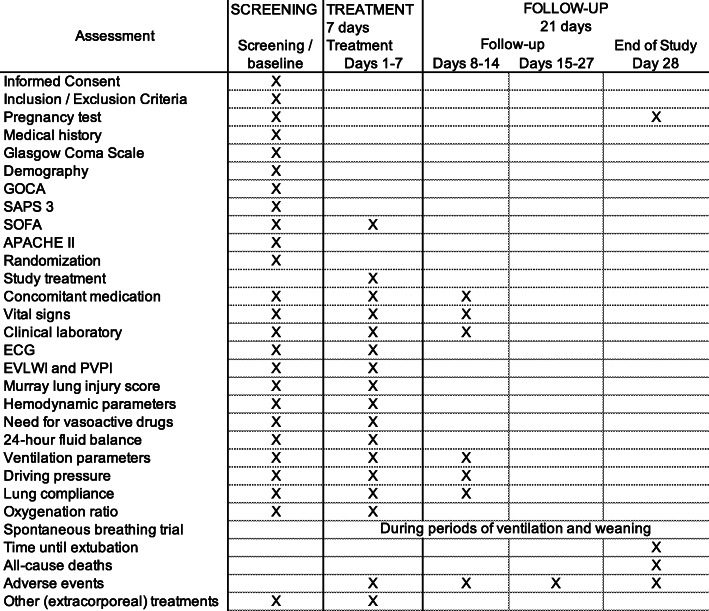
Schedule of activities

The intended timeline for each participant’s study activities is outlined in Fig. 2.

### Sample size {14}

Given that the primary objective of the study is to investigate the safety of solnatide, the study sample size of 95 randomized patients is based on feasibility criteria.

Patients will be randomized to 3 groups of 20 patients each, representing the 3 multiple ascending dose groups receiving solnatide at doses of 5 mg, 60 mg and 125 mg, and 35 patients will receiving placebo in total, with 15 patients receiving placebo in Dosing Group A (low dose) and Dosing Group B (middle dose) and 20 patients receiving placebo in Dosing Group C (high dose).

Nevertheless, considering the key preliminary efficacy endpoint and the results of a Phase IIa study (2012-001863-64), a sample size of 20 evaluable patients in each treatment group will have approximately 80% power to detect a difference in means between solnatide and placebo of 3.2 ml/kg in the change from baseline to day 7 of EVLWI with a common SD of 3.5 using a two-group *t*-test with a 0.05 two-sided significance level and no alpha level adjustment for multiple testing.

### Recruitment {15}

All ARDS patients in participating centers will be screened for eligibility. In case of slow or insufficient recruitment, the sponsor will consider increasing the number of study sites.

## Assignment of interventions: allocation

### Sequence generation {16a}, concealment mechanism {16b} and implementation {16c}

A randomization plan will be generated before study start. No stratification factors are planned. Three different randomization lists (one for each step of the study) will be prepared with the following randomization ratios:

- 2:1 in the first step of solnatide 5 mg vs. placebo
- 4:1 in the second step of solnatide 60 mg vs. placebo
- 4:1 in the third step of solnatide 125 mg vs. placebo

The randomization process will be managed electronically (block-wise generation of random numbers) by means of an Interactive Web Response System (IWRS) to ensure that treatment assignment is unbiased and concealed from patients and investigator staff. The results of the randomization are merely revealed to the responsible study pharmacist, who will then facilitate the preparation of the respective IMP. IMP vials are transferred to the investigators in a neutral container. Solnatide and placebo solutions cannot be distinguished from one another by the investigators.

## Assignment of interventions: blinding

### Who will be blinded {17a}

This is a double-blind study. Patients and all clinical team members involved in the study are blinded. Solnatide and saline solution have the same appearance. All personnel except pharmacy staff directly involved in the study, including the investigator, are blinded to the medication codes.

Pharmacy staff in charge of labelling and preparing the IMP will be unblinded. As soon as a patient is randomized, an email will automatically be sent to the pharmacist instructing him or her to prepare a solnatide or saline (placebo) solution for that randomization number. The syringe containing 5 ml of either one or the other solution will be appropriately labelled and delivered to the ICU for administration. The pharmacist will then print and sign the email for filing purposes. No clinical staff member has access to the treatment preparation instructions except for the study pharmacists. The trial statistician will be blinded during the analysis of the data.

### Procedure for unblinding if needed {17b}

Individual patient unblinding during the trial may occur in case of patient emergencies or medically important adverse events including cases of COVID-19.

The code breaking procedure is managed by means of an online-specific utility implemented in the eCRF and is available for the investigator and pharmacovigilance staff only. Sealed envelopes will also be provided as backup in the unlikely case of system unavailability.

If during the study the investigator needs to break a code (for emergency reasons), he/she has to follow the procedure implemented in the system clicking on ad hoc icon present on the toolbar.

In case of an individual patient unblinding, an automatic email will be sent to the study team notifying the occurrence of a code break reminding the investigator to discontinue the patient from the study.

## Data collection and management

### Plans for assessment and collection of outcomes {18a}

All data collected for this trial, e.g., eligibility criteria, demography, medical history, concomitant medication, physical examinations, vital sign measurements, laboratory results, and ECG data, is first recorded in site specific clinical charts based either on electronic or paper documents. If electronic charts are used, the electronic system should be compliant with applicable requirements. No study-related information will be stored on the eCRF only.

For laboratory tests, each site uses its own certified routine laboratory, which also provides its own reference values for each parameter obtained for study purposes. Measurements like ECG or hemodynamic parameters will be obtained using medical-grade, certified equipment.

Clinical scores are assessed based on the following resources:

- Gas exchange, Organ failure, Cause, Associated disease (GOCA) score [26, 27]
- Sequential Organ Failure Assessment (SOFA) [28]
- Acute Physiology and Chronic Health Evaluation (APACHE) II score [29]
- Simplified Acute Physiology Score (SAPS) 3 [30, 31]

### Plans to promote participant retention and complete follow-up {18b}

Not applicable as this is a study of patients with ARDS in intensive care and under mechanical ventilation.

### Data management {19}

Designated investigator staff will enter the data required by the protocol into the eCRF using fully validated software that conforms to 21 CFR Part 11 requirements as well as GAMP 5 and PIC/S requirements. Designated investigator site staff will not be given access to the Electronic Data Capture (EDC) system until they are trained.

Web-based software will be used and no installation procedure is needed. Each site will be authorized by the administrator to access the eCRF. Each site-qualified personnel will be allowed to access the eCRF by means of a “login mask” requiring user ID and password and may read, modify, and update only the information he/she previously reported. Each page reports site code and subject code.

Online validation programs will check for data discrepancies and, by generating appropriate error messages, allow the data to be confirmed or corrected before transfer to the CRO working on behalf of the sponsor. The investigator will certify that the data entered into the eCRF is complete and accurate.

After database lock, the investigator will receive a CD-ROM of subject data for archiving at the investigational site.

### Confidentiality {27}

Participant confidentiality and privacy is strictly held in trust by the participating investigators, their staff, and the sponsor(s) and their interventions. This confidentiality is extended to cover testing of biological samples and genetic tests in addition to the clinical information relating to participants. Therefore, the study protocol, documentation, data, and all other information generated will be held in strict confidence. No information concerning the study or the data will be released to any unauthorized third party without prior written approval of the sponsor.

All research activities will be conducted in as private a setting as possible.

The study monitor, other authorized representatives of the sponsor, representatives of the IRB/IEC, regulatory agencies, or pharmaceutical company supplying study product may inspect all documents and records required to be maintained by the investigator, including but not limited to medical records (office, clinic, or hospital) and pharmacy records for the participants in this study. The clinical study site will permit access to such records.

The study participant’s contact information will be securely stored at each clinical site for internal use during the study. At the end of the study, all records will continue to be kept in a secure location for as long a period as dictated by the reviewing IRB/IEC, institutional policies, or sponsor requirements.

Study participant research data, which is for purposes of statistical analysis and scientific reporting, will be collected and stored at the OPIS Data Center. This will not include the participant’s contact or identifying information. Rather, individual participants and their research data will be identified by a unique study identification number. The study data entry and study management systems used by clinical sites and by OPIS Data Center staff will be secured and password protected.

### Plans for collection, laboratory evaluation, and storage of biological specimens for genetic or molecular analysis in this trial/future use {33}

These are not applicable

## Statistical methods

### Statistical methods for primary and secondary outcomes {20a}

Data collected in this study will be listed and summarized as described below by treatment group. Data from all sites will be pooled and summarized. Continuous data will be summarized by mean, standard deviation (SD), median, first and third quartiles, minimum, and maximum. Categorical data will be presented by absolute and relative frequencies (n and %) or contingency tables. All statistical tables, listings, figures, and analyses will be performed by means of SAS® release 9.4 or later (SAS Institute, Inc., Cary, NC, USA).

A two-sided alpha level 0.05 will be considered. No alpha level adjustment will be made for secondary preliminary efficacy outcome variables and for multiple comparisons. Patients will be included in each analysis based on available assessments. No methodology for missing data handling will be applied, except for the EVLWI efficacy endpoint. Censoring rules for time-to-event analyses will be detailed in the Statistical Analysis Plan.

There is no primary efficacy endpoint as the primary objective of the study is to investigate the safety of solnatide. All efficacy analysis will be considered as secondary preliminary efficacy analyses. All secondary preliminary efficacy analyses will be performed based on the ITT set as primary analysis and on the PP set as supportive analysis and will be reported by planned treatment cohort.

No methodology for missing data handling will be applied for secondary preliminary efficacy endpoints, except for EVLWI. The homogeneity of treatment effects across different centers will not be tested.

#### Change from baseline to day 7 in extravascular lung water index (EVLWI)

The absolute change from baseline in extravascular lung water index (EVLWI) after 7 days from randomization, as assessed with a validated bedside measurement (single indicator transpulmonary thermodilution measurement, such as with PiCCO® from Pulsion or VolumeView from Edwards), will be analyzed by means of an analysis of covariance (ANCOVA) model with baseline value as covariate and treatment cohort as an independent class variable. Mean estimates will be provided, together with their corresponding two-sided 95% confidence intervals.

A hierarchical testing procedure will be carried out to control multiplicity, where statistical comparison will first be performed between the highest solnatide dose cohort and the placebo cohort. Statistical comparison between the next lower dose(s) and placebo will be performed only in case of statistical significance of the comparison between the highest solnatide dose cohort and the placebo cohort.

The analysis described above will be performed on data where the Last Observation Carry Forward approach is applied for handling missing data. Additional imputation methodologies for missing data may be applied as sensitivity analysis (further details to be provided in the Statistical Analysis Plan).

1. Secondary preliminary efficacy endpoints

#### Change from baseline to day 7 in Pulmonary Vascular Permeability Index (PVPI)

The absolute value and the change from baseline will be analyzed by treatment group for PVPI by means of summary descriptive statistics at each time point until day 7.

#### Lung compliance during mechanical ventilation

Static lung compliance is the change in volume for any given applied pressure and is derived as the ratio between tidal volume (*V*_t_) and the difference between plateau pressure and positive end-expiratory pressure (PEEP).

The absolute value and the change from baseline will be analyzed for static lung compliance by means of summary descriptive statistics at each time point through day 14, if patient is mechanically ventilated (controlled mechanical ventilation).

#### Murray lung injury score (LIS)

The absolute value and the change from baseline will be analyzed for Murray Lung Injury Score by means of summary descriptive statistics at each time point until day 7 if chest X-ray is available.

#### Sequential Organ Failure Assessment (SOFA)

The absolute value and the change from baseline will be analyzed for SOFA score by means of summary descriptive statistics at each time point until day 7.

#### Oxygenation ratio (P_a_O_2_/F_i_O_2_ ratio)

The absolute value and the change from baseline will be analyzed for oxygenation ratio (P_a_O_2_/F_i_O_2_) by means of summary descriptive statistics at each time point during first 7 days.

#### Ventilation parameters

The absolute value and the change from baseline will be analyzed for each ventilation parameter (ventilatory plateau pressure, *V*_t_, PEEP, respiratory rate, F_i_O_2_, PIP, mean airway pressure, peak airway pressure) by means of summary descriptive statistics at each time point through day 14 for patients mechanically ventilated (controlled mechanical ventilation, assisted breathing, non-invasive ventilation).

#### Driving pressure (*P*_plat_ − PEEP)

The absolute value and the change from baseline will be analyzed for driving pressure (*P*_plat_ − PEEP) by means of summary descriptive statistics at each time point through day 14 for patients mechanically ventilated (controlled mechanical ventilation, assisted breathing, non-invasive ventilation).

#### Spontaneous Breathing Trial

The time (in days) from the first spontaneous breathing trial to the first successful extubation / unassisted breathing will be calculated. Summary descriptive statistics will be provided.

In addition, patients will be classified according to the following outcome definitions [32]:

- Simple
- Successful SBT after the first attempt
- Difficult
- Failed SBT at first attempt and
- Required up to three trials or
- Required < 7 days to reach successful SBT
- Prolonged
- Required > 7 days to reach successful SBT

#### Time to extubation through day 28

Time to extubation (in days) is defined as the difference between the first extubation date and the randomization date. Patients still intubated at day 28 or who die while still intubated will be censored at day 28. Summary descriptive statistics will be provided for the time to extubation.

#### Ventilator-free days (VFD) through day 28

VFD is defined as the number of days from randomization to day 28 after achieving unassisted breathing for patients who maintained unassisted breathing for at least two consecutive calendar days. If a patient survived for more than 48 consecutive hours of unassisted breathing but required assisted breathing (for any reason) before day 28, the number of VFD is the number of days of successful unassisted breathing through day 28. All periods of successful unassisted breathing (> 48 consecutive hours) are taken into account. Unassisted breathing is defined as being extubated with face mask, with nasal prong oxygen or room air or T-tube breathing or tracheostomy mask breathing or CPAP breathing with ≤ 8 cm H_2_O pressure support of assisted ventilator support, according to weaning protocol. Patients who die before day 28 will be assigned the number of days of successful unassisted breathing. If a patient required more than 28 days of mechanical ventilation, the value for VFD will be set to 0.

The parameter of ventilator-free days will be analyzed by means of an analysis of variance (ANOVA) model with treatment cohort as an independent class variable. Mean estimates will be provided, together with their corresponding two-sided 95% confidence intervals.

No multiplicity adjustment for multiple comparison will be applied, given that statistical comparison will be performed only between the highest solnatide dose cohort and the placebo cohort. A statistical comparison between lower dose(s) and placebo will be performed only in case of statistical significance of the comparison between the highest solnatide dose cohort and the placebo cohort.

#### Days of hospitalization through day 28

Length of hospital stay (in days) is defined as the difference between the discharge date and the randomization date. Patients still in the hospital at day 28 or who die during hospitalization will be censored at day 28. Days of outpatient hospitalization will not be included. Summary descriptive statistics will be provided for the number of days of hospitalization.

#### Days of stay at ICU through day 28

Length of stay at ICU (in days) is defined as the number of calendar days a patient was in the ICU. Patients still in the ICU at day 28 or who die during the stay at ICU will be censored at day 28. Summary descriptive statistics will be provided for the number of days of stay at the ICU.

2. Safety analyses

Safety analyses will be conducted on the Safety Set and will be reported by actual treatment cohort. No methodology for missing data handling will be applied for safety parameters.

#### Drug-related adverse events through day 14

The incidence of treatment-emergent drug-related AEs through day 14 after randomization, both in terms of number of events and in terms of patients with at least one event, will be tabulated by MedDRA System Organ Class (SOC) and Preferred Term (PT).

#### All adverse events through day 28

According to the onset date of the event, AEs will be defined as follows:

- Treatment-emergent AEs are those events with an onset date after treatment initiation;
- Non-treatment-emergent AEs are those events with an onset date between screening and treatment initiation.

Non-treatment-emergent adverse events will be listed only. The incidence of treatment-emergent adverse events (TEAE) will be tabulated by MedDRA System Organ Class (SOC) and Preferred Term (PT). The incidence of TEAEs will also be summarized by system organ class, preferred term, and severity (based on investigator’s judgment).

The same analysis will be repeated for SAEs regardless of drug relationship, for drug-related SAEs, AEs with an outcome of death, and AEs leading to discontinuation of treatment. Deaths reportable as SAEs will be listed by patient and tabulated by MedDRA SOC and PT.

#### All-cause deaths through day 28

The number and proportion of deceased patients will be reported. In addition, the time (in days) from randomization to the event will be used for analysis. Subjects without an event will be censored at the earlier of the last contact date or day 28. The Kaplan-Meier estimates of the survival functions for each treatment cohort will be plotted and summarized.

#### Vital signs

The absolute value and the change from baseline will be analyzed for each parameter (including heart rate, systolic and diastolic blood pressure and body temperature) by means of summary descriptive statistics at each time point through day 14 after randomization.

#### ECG parameters

Evaluation of ECG data will be performed by an external provider.

The absolute value and the change from baseline will be analyzed for each cardiac parameter (PQ, QRS, QT, and QTc intervals [Fridericia’s formula] and heart rate) by means of summary descriptive statistics at each time point (first 7 days of study). Based upon these parameters, heart rhythm anomalies / conduction abnormalities and changes/abnormalities in ECG morphology will be assessed and summarized.

#### Laboratory parameters

The absolute value and the change from baseline will be analyzed for each laboratory parameter (separately for hematology, clinical chemistry, blood gases, and urinalysis) by means of summary descriptive statistics at each time point through day 14. In addition, shift tables using the low/normal/high classification according to the laboratory normal ranges to compare baseline to the worst on-treatment value will be provided.

Listings of all laboratory data with values flagged to show the classifications relative to the laboratory normal ranges will also be generated.

#### Twenty-four-hour fluid balance

The absolute value and the change from baseline will be analyzed for the 24-h fluid balance parameter by means of summary descriptive statistics at each time point through day 7. Twenty-four-hour fluid balance at a given timepoint will be determined as the difference, at the timepoint, between the complete amount of fluid supplied to the patient over 24 h and the complete amount of fluid lost by the patient over the same period.

#### Hemodynamic parameters

The absolute value and the change from baseline will be analyzed for each hemodynamic parameter (i.e., mean arterial pressure, pulmonary blood volume (PBV), cardiac index and cardiac output) by means of summary descriptive statistics at each time point until the end of treatment.

#### Need for vasoactive drugs

The number and proportion of patients who required vasoactive drugs at any time through the end of treatment will be reported.

3. Baseline descriptive statistics

Patient demographics and baseline characteristics will be summarized on the ITT set, overall and by treatment cohort, and by means of summary descriptive statistics. A complete description of subject disposition will be provided, overall and by treatment cohort specifying the number of randomized subjects, number of subjects at each visit, and completed and discontinued subjects and the reason for the discontinuation. The analysis populations will be described and the reasons for excluding the subject from any analysis set will be provided with the number of protocol violators per each criterion. Medical history data will be presented by MedDRA System Organ Class and Preferred Term.

4. Treatments

The Safety Set will be used for the following analyses.

#### Investigational treatment

Duration of exposure to study treatment, defined as the time (days) elapsed from the date of the first treatment administration to the date of the last treatment administration and number of administrations will be summarized. Cumulative dose (mg), defined as the total dose given during treatment exposure, will be summarized. The number of patients with dose interruptions/permanent discontinuation will be presented along with the reasons for the dose interruptions/discontinuation.

#### Concomitant treatments

Concomitant medications or procedures taken concurrently with the study treatment will be listed and summarized by WHO Anatomical Therapeutic Chemical (ATC) Class and Preferred Term. These summaries will include medications starting on or after the start of study treatment, or medications starting prior to the start of study treatment and continuing after the start of study treatment. Prior medications starting and ending prior to the start of study treatment will be listed only.

### Interim analyses {21b}

No formal interim efficacy analysis is planned.

### Methods for additional analyses (e.g., subgroup analyses) {20b}

Additional subgroup analyses will be detailed in the Statistical Analysis Plan.

### Methods in analysis to handle protocol non-adherence and any statistical methods to handle missing data {20c}

Patients will be included in each analysis based on available assessments. No methodology for missing data handling will be applied, except for the EVLWI efficacy endpoint.

### Plans to give access to the full protocol, participant-level data, and statistical code {31c}

These are not applicable.

## Oversight and monitoring

### Composition of the coordinating center and trial steering committee {5d}

The clinical study is coordinated by a professional Clinical Research Organization (CRO). The CRO and the sponsor form the trial steering committee.

The data management is part of the responsibility of the CRO and cannot accessed by the sponsor. No unblinded data are submitted from the data management to the sponsor and to the investigators.

There is no endpoint adjudication committee foreseen.

### Composition of the data monitoring committee, its role and reporting structure {21a}

The sponsor has appointed an independent Data and Safety Monitoring Board. The DSMB’s role is defined in the DSMB Charter of clinical trial EudraCT 2017-003855-47 of March 2018. The DSMB is composed of individual experts in the field of ARDS from the following countries: Germany, Spain, USA. All members of the DSMB have no competing interest in the trial.

The DSMB is responsible for reviewing the safety and tolerability after completion of each dose group and confirmation in writing that the study can proceed with the next higher dose group (dose escalation). After completion of the study, the DSMB will make a final statement concerning overall safety (the primary objective of the study). Overall safety will be assessed in a final session of the DSMB by a summarizing review of the incidence of mortality, adverse events, and serious adverse events, as well as by analysis of relevant laboratory data and ECGs. The DSMB will provide advice to the Sponsor which will be responsible for promptly reviewing the DSMB recommendations, deciding whether to continue, modify, or terminate the trial; and determining whether amendments to the protocol or changes in study conduct are required.

The DSMB is required to act in accordance with the ethical principles derived from the Declaration of Helsinki.

Communication with DSMB members will be primarily through an independent statistician not involved in the management and performance of this study. It is not expected that study investigators or co-investigators will directly communicate with DSMB members.

### Adverse event reporting and harms {22}

All identified non-serious AEs (related and unrelated) must be recorded and described on the non-serious AE page of the eCRF.

#### Serious adverse event reporting

Every SAE, regardless of suspected causality, occurring after screening and until at least 28 days after randomization must be reported to the sponsor within 24 h of site awareness. Any SAE experienced after this 28-day period should only be reported to the sponsor if the investigator suspects a causal relationship to the study treatment. Recurrent episodes, complications, or progression of the initial SAE must be reported as follow-up to the original episode within 24 h of the investigator receiving the follow-up information. Information about all SAEs will be recorded on the AE page of the eCRF and transmitted through the SAE tool. In case of technical difficulties, SAE notification can be carried out by sending a paper SAE form to the Pharmacovigilance Officer via email or by fax.

All SAEs still ongoing at end of study will be followed up by means of queries requesting updates. Other supporting documentation of the event may be requested by the study sponsor and should be provided as soon as possible. The study sponsor will be responsible for notifying Health Authorities of any unexpected fatal or life-threatening suspected adverse reaction as soon as possible, but in no case later than 7 calendar days after the sponsor’s initial receipt of the information.

Suspected unexpected serious adverse reactions (SUSARs) will be collected and reported to the competent authorities and relevant ethics committees in accordance with Directive 2001/20/EC or as per national regulatory requirements in participating countries.

#### Reporting events to participants

Should an event occur that changes the overall benefit/risk ratio of the study, the Sponsor shall evaluate if a risk minimization measure is needed. Should this measure require a substantial amendment to the protocol, the informed consent and patient information will be revised and submitted to the patient for written consent.

### Frequency and plans for auditing trial conduct {23}

Clinical site monitoring will be conducted to ensure that the rights and wellbeing of human subjects are protected, that the reported study data are accurate, complete, and verifiable, and that the conduct of the study complies with the currently approved protocol, with Good Clinical Practice (GCP), and with applicable regulatory requirements. Monitoring for this study will be performed by a Contract Research Organization (CRO), OPIS. Details of clinical site monitoring are documented in a Monitoring Plan (MP). The MP describes in detail who will conduct the monitoring, at what frequency monitoring will be done, at what level of detail monitoring will be performed, and the distribution of monitoring reports. Further audits are at the discretion of the respective authorities in charge of overseeing medicinal products trials.

### Plans for communicating important protocol amendments to relevant parties (e.g., trial participants, ethical committees) {25}

Important protocol modifications will be reported to the responsible ethics committees and, after approval, made accessible to the study centers. As far as the ICF is subject to any revision, all future participants will be provided with an updated version of the ICF. Protocol amendments will not be communicated to past participants.

## Dissemination plans {31a, b, c}

This study will ensure that the public has access to the published results of the research. The International Committee of Medical Journal Editors (ICMJE) member journals have adopted a clinical trials registration policy as a condition for publication. The ICMJE defines a clinical trial as any research project that prospectively assigns human subjects to intervention or concurrent comparison or control groups to study the cause-and-effect relationship between a medical intervention and a health outcome. Medical interventions include drugs, surgical procedures, devices, behavioral treatments, process-of-care changes, and the like. Health outcomes include any biomedical or health-related measures obtained in patients or subjects, including pharmacokinetic measures and adverse events. The ICMJE policy requires that all clinical trials be registered in a public trials registry such as ClinicalTrials.gov, which is sponsored by the National Library of Medicine. Other biomedical journals are considering adopting similar policies.

Access to participant-level datasets and statistical code can be granted upon request to the sponsor’s discretion.

## Discussion

This protocol describes the process of a Phase II clinical trial investigating the safety of a novel inhaled agent for the treatment of moderate to severe ARDS. In addition to the challenges one faces with every pharmaceutical investigation, this study has some peculiarities which shall be discussed here briefly.

The route of administration of the IMP is bronchial inhalation. One major advantages of this way of administration is the possibility to bring the IMP straight to the desired site of action (i.e., the lungs) with minimal plasmatic absorption, depending on the agent’s pharmacokinetic properties. The latter was shown for solnatide in a first-in-man study [22], which may indicate a low risk of undesired systemic effects. However, inhalative administration, unlike intravenous or oral use, is fraught with some degree of variation in terms of the amount of agent that actually reaches the alveolae. This is due to each individual’s anatomical and physiological features of the respiratory tract and has been addressed systematically in earlier research [33]. To minimize this effect as best as possible, standardization of the procedures is key. Therefore, all patients in this study will be treated using the same model of bronchial inhaler and great care was taken to make the setup of the respirator, tubes, suction devices, and tubes as similar as possible between patients and study centers.

Another issue to keep in mind is the relatively narrow timeframe for inclusion of a patient. The protocol requires a diagnosis of moderate to severe ARDS not older than 48 h, yet the patient needs to be stable in this condition for at least 8 h. In practice, potential study patients will initially often be treated in peripheral hospitals, deteriorate there until they meet the required criteria, and then be transferred to one of the study centers for further treatment in a more or less unstable condition. Therefore, once the patient is stable enough to be enrolled in the trial, there will possibly be only little time left to get informed consent and have all data necessary for inclusion ready. In order to respond to this challenge, proper preparation of the required examinations and procedures is advised. Also, determination of the exact point in time of the ARDS diagnosis is key. Respiratory deterioration alone does not fulfill the Berlin criteria definition of ARDS, so technically, the 48 h from diagnosis until possible enrolment will frequently not start until appropriate chest imaging was performed.

The patient’s possible need for extracorporeal membrane oxygenation (EMCO) therapy during the course of the intervention phase of the trial should also be considered early on. While ECMO therapy at screening leads to exclusion of the patient, once a patient is enrolled, extracorporeal therapy will be initiated as is clinically appropriate. This is, for ethical reasons, not to be disputed. However, quality of the collected data will be compromised in patients on ECMO therapy during the first 7 days of the trial. In particular, extravascular lung water (index) was found to be estimated significantly higher in such patients due to the altered hemodynamics caused by the extracorporeal circuit [34]. The responsible investigator will have to make an individual decision for every patient about to be included in the trial, considering both quality of data on the one hand and potential selection bias against the most severely sick patients on the other hand.

A last point to consider might be the fact that basically all patients will be unable to give informed consent themselves at the time of screening. Inclusion criteria demand that any patient needs to be mechanically ventilated at screening, and this will usually go along with the use of some form of sedative medication. So, having the procedures for enrolling such patients in place according to local legal requirements must be taken care of beforehand. Moreover, it is assumed that patients will be hospitalized in the study facility over the entire course of the observational period due to the severity of their conditions. In case a patient is transferred sooner than 28 days after enrolment, individual measures need to be found in order to ensure data assessment.

We present here the protocol for a randomized, double-blind, parallel-group clinical trial investigating the safety of a novel inhaled agent for the treatment of pulmonary permeability edema in patients with moderate to severe ARDS. As was laid out, especially during screening and the intervention phase (days 0 to 7), the coordination of tasks and procedures can be demanding at times. However, with proper preparation of the required processes and some consideration of the possible pitfalls discussed above, reliable, high-quality data can be collected throughout the 28-day intervention and follow-up period.

## Trial status

Current protocol version: 8.0, 2 August 2021

Start of recruitment: 23.05.2018

Approx. date of completion: start + 4 years

## Data Availability

Only the designated trial investigators will have access to the personal data of participants and to the final data set. The original eCRF pages generated during the study will become the property of the sponsor.

## Abbreviations

AE: Adverse event
ALC: Alveolar liquid clearance
ANCOVA: Analysis of covariance
ANOVA: Analysis of variance
APACHE: Acute Physiology and Chronic Health Evaluation (score)
ARDS: Acute respiratory distress syndrome
ATC: Anatomical therapeutic chemical
ATP: Adenosine triphosphate
AUC: Area under the curve
BMI: Body mass index
BW: Body weight
CD-ROM: Compact disc – read-only memory
*C*_max_: Maximum concentration
COPD: Chronic obstructive pulmonary disease
COVID-19: Coronavirus disease 2019
CPAP: Continuous positive airway pressure
CRO: Contract research organization
DSMB: Data safety monitoring board
ECG: Electrocardiogram
ECMO: Extracorporeal membrane oxygenation
eCRF: Electronic case report form
EDC: Electronic data capture
ENaC: Epithelial sodium channel
EVLWI: Extravascular lung water index
F_i_O_2_: Inspiratory fraction of oxygen
GCP: Good clinical practice
GOCA: Gas exchange, Organ failure, Cause, Associated disease (score)
ICH: International Council for Harmonisation of Technical Requirements for Pharmaceuticals for Human Use
ICMJE: International Committee of Medical Journal Editors
ICU: Intensive care unit
IEB: Independent ethics committee
IMP: Investigational medicinal product
IRB: Institutional review board
ITT: Intention-to-treat
IWRS: Interactive web response system
LIS: Murray lung injury score
MAP: Mean arterial pressure
MLC: Myosin light chain
NO: Nitrogen oxide
NOAEL: No observed adverse effect level
P_a_CO_2_: Arterial carbon dioxide partial pressure
P_a_O_2_: Arterial oxygen partial pressure
PBV: Pulmonary blood volume
PEEP: Positive end-expiratory pressure
PGD: Primary graft dysfunction
PKC: Protein kinase C
PP: Per-protocol
PVPI: Pulmonary vascular permeability index
ROS: Reactive oxygen species
SAE: Serious adverse event
SAPS: Simplified Acute Physiology Score
SBT: Spontaneous breathing trial
SD: Standard deviation
SO_2_: Oxygen saturation
SOFA: Sequential Organ Failure Assessment
SUSAR: Suspected unexpected serious adverse reaction
TEAE: Treatment-emergent adverse event
TNF-m: Tumor necrosis factor e
VFD: Ventilator-free days
*V*_t_: Tidal volume
WHO: World Health Organization
